# *Bacillus* As Potential Probiotics: Status, Concerns, and Future Perspectives

**DOI:** 10.3389/fmicb.2017.01490

**Published:** 2017-08-10

**Authors:** Fouad M. F. Elshaghabee, Namita Rokana, Rohini D. Gulhane, Chetan Sharma, Harsh Panwar

**Affiliations:** ^1^Department of Dairy Science, Faculty of Agriculture, Cairo University Giza, Egypt; ^2^Department of Dairy Microbiology, College of Dairy Science and Technology, Guru Angad Dev Veterinary and Animal Sciences University Ludhiana, India

**Keywords:** spore formers, *Bacillus*, beneficial microbes, probiotics, intestinal microbiota, human health, mechanism of action

## Abstract

Spore-forming bacilli are being explored for the production and preservation of food for many centuries. The inherent ability of production of large number of secretory proteins, enzymes, antimicrobial compounds, vitamins, and carotenoids specifies the importance of bacilli in food chain. Additionally, *Bacillus* spp. are gaining interest in human health related functional food research coupled with their enhanced tolerance and survivability under hostile environment of gastrointestinal tract. Besides, bacilli are more stable during processing and storage of food and pharmaceutical preparations, making them more suitable candidate for health promoting formulations. Further, *Bacillus* strains also possess biotherapeutic potential which is connected with their ability to interact with the internal milieu of the host by producing variety of antimicrobial peptides and small extracellular effector molecules. Nonetheless, with proposed scientific evidences, commercial probiotic supplements, and functional foods comprising of *Bacillus* spp. had not gained much credential in general population, since the debate over probiotic *vs* pathogen tag of *Bacillus* in the research and production terrains is confusing consumers. Hence, it’s important to clearly understand the phenotypic and genotypic characteristics of selective beneficial *Bacillus* spp. and their substantiation with those having GRAS status, to reach a consensus over the same. This review highlights the probiotic candidature of spore forming *Bacillus* spp. and presents an overview of the proposed health benefits, including application in food and pharmaceutical industry. Moreover, the growing need to evaluate the safety of individual *Bacillus* strains as well as species on a case by case basis and necessity of more profound analysis for the selection and identification of *Bacillus* probiotic candidates are also taken into consideration.

## Introduction

The interest in the field of beneficial microbes has emerged multiple folds since its inception by the Russian Noble laureate, Elie Metchnikoff. The term Probiotics, taken as an un-challenged synonym to beneficial microbes, has gained popularity over the years and has found application in several general health and clinical scenarios. Probiotics are *live microorganisms, which when administered in adequate amounts confer health benefits to the host* (FAO/WHO, 2002). Probiotic formulations are being developed and standardized for both human and animal consumption. Different dairy/functional foods/dietary supplements and pharma formulations harbor probiotic strains, intended for various health benefits in humans. Probiotics have also found application in animal feed for prevention of gastrointestinal infections, with extensive use in the poultry and aquaculture industries (Hong et al., 2005). The consumer awareness, search for alternate, safe and cost-effective treatments, and concern of developing antibiotic resistance has compelled researchers to find an alternate to ongoing therapeutic regimes, mainly dependent over antibiotics. Among the large number of suggested options, probiotic therapy seems to be the most viable one, with long history of consumption and assured safety. LAB and *Bifidobacterium* spp. are the two globally recognized groups of bacteria that are being consumed for their potential health benefits. Other preferred bacteria include strains of *Enterococcus, Streptococcus* and *Bacillus* spp. (Majeed et al., 2016); along with few strains of *Saccharomyces* spp. Several reference probiotic strains have been shown to play a potential role in management of several clinical scenarios *viz*. diarrhea, inflammatory bowel disease, obesity, type 2 diabetes, cardiovascular disease, cancer etc. (Mallappa et al., 2012; Panwar et al., 2014, 2016; Ranji et al., 2015). It has been clearly understood that the gut inhabitants and their proper balance is the foremost criteria that determines healthy status, particularly in terms of metabolic disorders (Wang et al., 2015). Research updates from several *in vivo* (Ellekilde et al., 2014) and human clinical trials (Shimizu et al., 2013; Scott et al., 2015) supports the hypothesis that any strategies targeting the re-customization of the gut inhabitants can help in reverting back to normal healthy phenotype.

**FIGURE 1:**
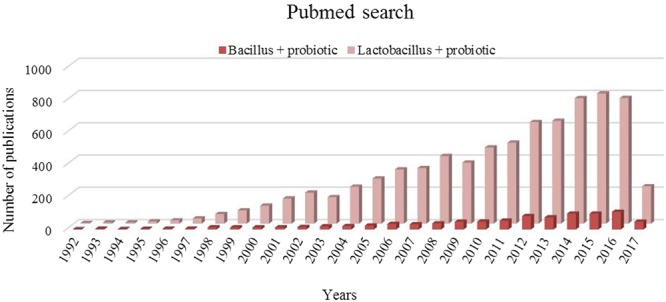
Pubmed trends for key words “*Bacillus*+ probiotic” and “*Lactobacillus* + probiotic” for last 25 years.

Besides the commonly explored strains, bacterial spore formers, mostly of the genus *Bacillus* do carry probiotic attributes. The value of non-spore former LAB for the maintenance of human and animal health has been acknowledged both scientifically in terms of published research data and commercially in form of the availability of probiotic products. However, in comparison to LAB, bacterial spore formers have not gained high popularity, particularly in terms of research interest (**Figure 1**). Several *Bacillus* strains have been screened for their potential probiotic functionalities, in several *in vitro* and *in vivo* models. Besides qualifying the mandatory bench marks for a candidate probiotic; *Bacillus* spp. offers higher acid tolerance and better stability during heat processing and low temperature storage (Bader et al., 2012). Additionally, they have also been shown to possess pathogen exclusion, anti-oxidant, antimicrobial, immuno-modulatory (Lefevre et al., 2015; Shobharani et al., 2015; Ripert et al., 2016) and food fermentation (Terlabie et al., 2006) abilities.

Furthermore, scientific reports supported with evidence of safe use and long history of consumption supports the candidature of spore formers as potential probiotics and as functional food supplements due to their significant capacity of production of extracellular enzymes. *Bacillus* spp. has been used for production of food grade amylase, glucoamylase, protease, pectinase and cellulase in varying food stuffs (Ghani et al., 2013; Ouattara et al., 2017). Different species of *Bacillus* has also been used for the production of additional nutraceuticals including vitamins (e.g., riboflavin, cobalamin, inositol) and carotenoids for the synthesis of several health supplements for human consumption (Mohammed et al., 2014; Tanaka et al., 2014; Takano, 2016). Nevertheless, despite above benefits, these strains have not gained much importance and attention in current functional food industry due to their relatedness with few human pathogens. Few of the members of *Bacillus* spp. particularly, *B*. *cereus, B*. *weihenstephanensis, B*. *anthracis*, and *B. thuringiensis* species are known to produce various toxins, including ematic or enterotoxin (Cereulide), Bipartite exotoxins: protective antigen- lethal factor (PA-LF) and PA-edema factor (PA-EF), Cry and Cyt. Among them, Cereulide produced by *B*. *cereus* and *B*. *weihenstephanensis* is a major cause of food borne intoxications; while PA-LF and PA-EF are *B*. *anthracis* generated toxins associated with deadly illness in humans and animals.

Thereby, to understand the nature of beneficial spore former probiotic strains, their probiotic potential and safety concerns are important; not only because of their complexity and behavior in human GIT, but also due to their allochthonous (free living) nature, questioning their ability to colonize in the human gut (Hong et al., 2005). Though, the members of *Bacillus* genus have been consumed in the form of fermented foods since long time (Tamang et al., 2016), concerns regarding their safety are also raised. This review highlights the probiotic candidature of spore forming *Bacillus* spp. and presents an overview of the proposed health benefits, including application in food and pharmaceutical industry. The associated safety and licensing issues that influence the use of *Bacillus* spp. for commercial development has been summarized, together with evidence showing the growing need to evaluate the safety of individual *Bacillus* strains as well as species on a case by case basis.

## Niche and Availability of *Bacillus* Probiotics

**FIGURE 2:**
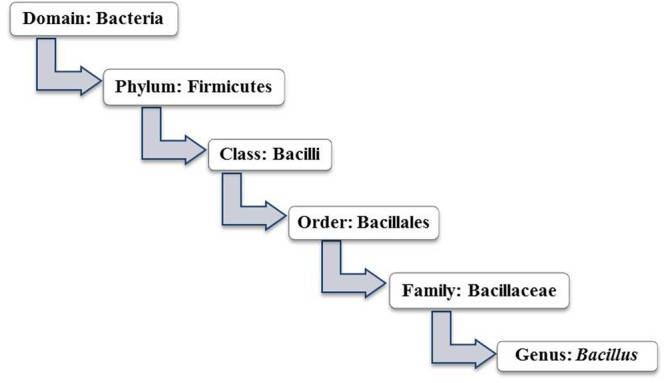
Taxonomy of genus *Bacillus* (Source: Bergey’s manual of systematic bacteriology: volume 3: The Firmicutes).

*Bacillus* signifies a Gram-positive, rod shaped, spore-forming, aerobic or facultative anaerobic bacterium. In general, the genus *Bacillus* is designated as a group of soil inhabitants. However, *Bacillus* spp. can be isolated from varied sources including air, water, human and animal gut, and also from vegetables and food (Alou et al., 2015; Kotb, 2015). Nevertheless, *Bacillus* spp. represents the most heterogeneous group in terms of phenotypic and genotypic characters. Some distinct species have also been recognized as opportunistic pathogen or toxin producer in human or animal hosts. The genus *Bacillus* is closely related to *Lactobacillus* spp., the distinguished candidate probiotic. Both share the same class, Bacilli under the phylum Firmicutes (**Figure 2**).

Looking toward the probiotic prospective, it is proclaimed that the candidate probiotic should be isolated from the gut of the target population, which helps them to thrive well within the gut. However, elementary attributes of native flora for survivability are not essential for spore-former(s). *Bacillus* spores can survive in extreme acidity of stomach, and tolerate bile salts and other hostile conditions of GIT. Besides, bacilli are more stable during processing and storage of food and pharmaceutical preparations, which make them more suitable ingredient for health promoting formulations. Thereby, in addition to human sources, *Bacillus* strains with probiotic attributes are also isolated from fermented or unfermented food sources (Adewumi et al., 2014; Rao et al., 2015) and are commercialized in the form of diverse range of health supplements (**Table 1**).

**Table 1:** Examples of probiotic supplements containing *Bacillus* spp. available in global market.

Product	Country	Components
Nutrition essentials Probiotic	United States	*Bacillus coagulans* 15B and fructooligosaccharide
NutriCommit	United States	*Bacillus subtilis, Bacillus coagulans*
Flora3	United States	*Bacillus coagulans, Saccharomyces boulardii*, and FOS
LifeinU^TM^	Europe	*Bacillus subtilis* CU1
THORNE	United States	*Bacillus coagulans*
Sunny Green Cleansing Green	United States	*Bacillus coagulans*
Just Thrive	United States	*Bacillus indicus* HU36, *Bacillus coagulans, Bacillus clausii, Bacillus subtilis* HU58
Vital Probiotics	United States	*Bacillus subtilis, L*. *rhamnosus, L*. *casei, Bifidobacterium longum, L*. *acidophilus, L*. *plantarum, Bifidobacterium breve*
MegaSporeBiotic	United Kingdom	*Bacillus indicus, Bacillus subtilis, Bacillus coagulans*
		*Bacillus licheniformis, Bacillus clausii*
Bio-Kult	United Kingdom	*Bacillus subtilis* PXN 21, *Lactobacillus, Bifidobacterium*, and *Streptococcus* strains
Enterogermina^®^	Europe	*B. clausii*
BioPlus 2B^®^	Denmark	*B. subtilis* CH201/DSM5749 and *B. licheniformis* CH200/DSM5749
GanedenBC^30^	United States	*B. coagulans*
Anaban^TM^	Europe	*B. subtilis*
Biosporin^®^	Europe	*B. subtilis, B. licheniformis*

## Spore-Formers In Human Gut

It is believed that *Bacillus* spp. is not a natural inhabitant of gut. They get colonized in to the intestinal tract after consumption of vegetables or raw food materials contaminated with soil microflora. Moreover, ingestion of fermented cereals or beans, such as Iru and Natto also make their way in to the intestine. Findings from *in vitro* studies claim that vegetative cells and spores of *B. cereus* can well defend the GIT stress and adhere to the intestinal epithelium. However commensal gut microbiota possesses inhibitory activity against them (Berthold-Pluta et al., 2015). A study by Tam et al. (2006) demonstrated that spores of *Bacillus* spp. could readily be recovered in the range of 10^3^-10^8^cfu/g of human feces. The 16S rRNA gene phylogenetic analysis of isolates demonstrated the presence of 10 different *Bacillus* species in examined fecal samples of 30 volunteers (Tam et al., 2006). Hoyles et al. (2012) further studied the diversity of *Bacillus* spp. and related spore-former bacteria in human feces and documented that the majority of recovered isolates belonged to Bacillaceae family. Two species, *B. clausii* and *B. licheniformis* were recovered most frequently. To find out the stage of *Bacillus* in human gut, Casula and Cutting (2002) targeted a genetically engineered chimeric gene, *ftsH-lacZ*, which is selectively and strongly expressed in the vegetative cells of *Bacillus subtilis* and reported their presence throughout the GIT. They stated that the spores germinated in significant numbers in the jejunum and ileum, suggesting their colonization into the small intestine. Recently, Ghelardi et al. (2015) documented that the orally administered *Bacillus* spp. follows transient colonization in to the intestine. Nevertheless, the impact of allochthonous *Bacillus* strains on the profile of fecal flora of the host during transition period has been proven significant. Nyangale et al. (2014) observed that after 28 day treatment of *Bacillus coagulans* in elderly subjects, baseline populations of *Faecalibacterium prausnitzii, Clostridium lituseburense* and *Bacillus* spp. were significantly higher, relative to the placebo group. Likewise, a study by Adami and Cavazzoni (1999) in piglet model has also shown that the feeding of *Bacillus coagulans* CNCM I-1061 increased aerobic and anaerobic sporeformers, decreased lactococci, enterococci, anaerobic cocci, and fecal coliforms in the treatment group.

## Spore-Formers In Food Chain

The consumption of vegetative cells and spores of *Bacillus* spp. by human beings is frequent through fermented foods and raw vegetables. A diverse range of *Bacillus* species are found to be associated to the natural fermentation of soy, locust been, maize, rice and many more substrates. For example, Natto (Japan), Gari (Africa) TapaiUbi (Malaysia), Douchi (China), Rabadi (India, Pakistan), Soibum (India), Ugba (Nigeria) etc. are among the popular functional foods naturally harboring the blend of *Bacillus* spp. and LAB. These fermented products exhibit unique sensory attributes, probably due to the activity of extracellular carbohydrate and protein degrading enzymes of *Bacillus* spp. origin (**Table 2**). The palatability and health promoting characteristic of these locally produced supplements has also attracted the attention of global market. A diverse range of LAB and *Bacillus* spp. isolated from such indigenous foods has been studied and are being used for the commercial preparations of functional products (Angmo et al., 2016; Von Mollendorff et al., 2016). Respectively, the strains of *B. subtilis, B. subtilis* var. natto, *B. clausii, B. licheniformis*, and *B. coagulans* etc. are being utilized to improve the quality and demand of functional foods globally (Beaumont, 2002; Chantawannakul et al., 2002; Inatsu et al., 2006).

**Table 2:** List of *Bacillus* species used for production of enzymes.

Class of enzyme	Name of enzymes	Enzyme producing *Bacillus* species
Carbohydrate degrading	α-Amylase, β-Amylase, Arabinase, Cellulase, Chitinase, Chitosanase, Dextranase, Galactanase,	*B*. *coagulans, B*. *subtilis, B*. *licheniformis, B*. *amyloliquifaciens, B*. *cereus, B*. *megaterium, B*. *caldolyticus, B*. *polymyxa, B*. *pumilus, B. circulans, B*. *firmus, B*. *brevis, B*. *macerans, B*. *stearothermophilus*
	β-1,3-glucanase, β-1,6-glucanase, Isoamylase, Lichenase, Levansucrase, Maltase, Mannanase, Pactate lysase, Phosphomannase, Pullulanase, Xylanase, Glucose isomerase	
Proteases	Aminopeptidase, Esterase, metal proteases, Serine proteases	*B*. *subtilis, B*. *cereus, B*. *licheniformis, B*. *amyloliquifaciens, B. megaterium, B*. *polymyxa, B*. *thermoproteolyticus, B. thuringiensis, B. pumilus*
Lipase	Phospholipase C, Thiaminase	*B*. *licheniformis, B*. *cereus, B*. *anthracis, B. thuringiensis, B. thiaminolyticus*
Nucleases	Doxyribonuclease, Ribonucleases, 3-nucleotidases, 5-nucleotidases	*B*. *amyloliquifaciens, B*. *subtilis, B*. *cereus, B*. *megaterium, B*. *pumilus*
Phosphatases	Alkaline phosphatase	*B*. *amyloliquifaciens, B*. *subtilis, B*. *cereus*
Other	β-lactamase,	*B*. *subtilis, B*. *cereus, B*. *anthracis, B*. *licheniformis, B*. *megaterium*
	Endo-N-acetylglucosaminidase,	
	Exo-N-acetylglucosaminidase	
	Endo-N-acetymuraminidase,	
	Exo-N-acetylglucosaminidase	

*Bacillus* strains are also getting recognition as potential probiotics which could promote human health by direct consumption of high concentrations of viable number of cells (Abdhul et al., 2015; Manhar et al., 2016). Several *Bacillus* probiotic strains have been approved by regulatory agencies of different countries for human use and are popular as general health promoting pharmaceutical formulations in global market (**Table 1**). So far commercial products of *Bacillus* composing functional foods are not popular in nutraceuticals market because the debate over probiotic *vs.* pathogen tag of *Bacillus* spp. is persisting in the research and production terrains. Hence, it’s important to clearly understand the phenotypic and genotypic characteristics of selective *Bacillus* spp. and their substantiation with those having GRAS status, to reach a consensus over the same.

## Genus *Bacillus*: Probiotic Or Pathogen

*Bacillus* spp. has received considerable taxonomic attention because of its economic as well as medical importance. The genus *Bacillus* has undergone considerable taxonomic changes over time. The number of species allocated to this genus increased to 318 in the “List of prokaryotic names with standing in nomenclature”^1^. With the advent of molecular taxonomy, Ash et al. (1991) separated 51 distinct *Bacillus* species into five phylogenetic clusters. In this review, we are looking over the significance and approach of *Bacillus* probiotics in current scenario. Thus, it will also be interesting to take note of the evolutionary connections of probiotic *Bacillus* species/strains with other members of the genera. According to Ash et al. (1991), group 1 of *Bacillus* forms the largest cluster of 28 species. Evolutionary distance tree showed specific relation between species, for example, there were three distinct clades of closely related species. First clade included *B. subtilis, B. atrophaeus, B. amyloliquifaciens, B. lautus, B. lentimorhus, B. licheniformis, B. popilhe*, and *B. pumilus.* Second and third clades comprised of *B. anthracis, B. cereus, B. meduso, B. mycoides, B. thuringiensis, B. maroccanus, B. simplex*, and *B. psychrosaccharolyticus.* However, all remaining members of the group did not show any significant relationship with them. Group 2, 3, 4, and 5 consisted of 7, 10, 2, and 3 species respectively. Two species, *B. alcalophilus* and *B. aneurinolyticus* remained ungrouped and formed a separate line of descent. Interestingly, the available data points out that both the probiotic *Bacillus* species (e.g., *B. subtilis, B. coagulance, B. licheniformis*, and *B. megaterium*) and potential human pathogens (*B. anthracis* and *B. cereus*) fall into group 1; however, both the forms are separated into distinct clans. Therefore, more deep analysis of the taxonomic positions of these species is needed to reach a definite conclusion.

**FIGURE 3:**
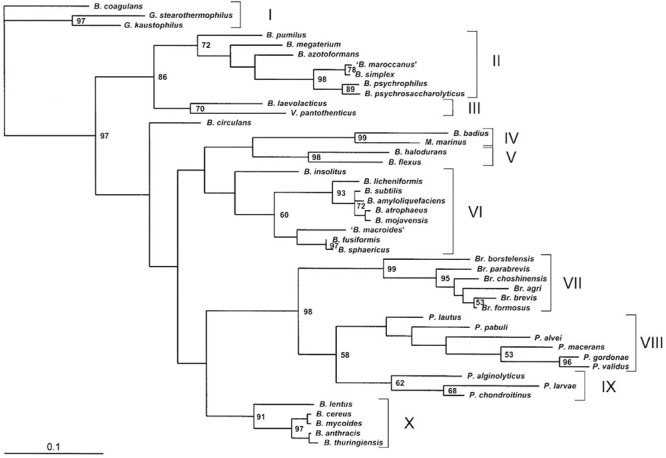
Phylogenetic relationships of 46 *Bacillus* species. The bar represents the unit length of the number of nucleotide substitutions per site (adopted from Xu and Cote, 2003).

In this context, Xu and Cote (2003) made an effort to determine the phylogenic relationship among 40 *Bacillus* species using nucleotide sequences of the 16S rDNA and the 16S–23S internal transcribed spacer (ITS). In this study, comparative sequence analysis of the 16S-23S ITS sequences, revealed 10 distinct phylogenetic clusters. Twenty-six evaluated *Bacillus* species were separated in seven groups; with groups II, V, VI, and X comprising of seven, two, nine and five *Bacillus* species, respectively (**Figure 3**). *Bacillus subtilis*, the type species fall in Group VI along with *B. amyloliquefaciens, B. atrophaeus*, and *B. mojavensis*. *B. coagulans* was placed in group I; *B. maroccanus* and *Bacillus simplex* in Group II; and *B. anthracis, B. cereus, B. mycoides*, and *B. thuringiensis* in Group X. This study revealed that two major pathogenic spp. of *Bacillus* (*B. anthracis* and *B. cereus*) fall into the Xth group, which is apparently unrelated to the *Bacillus* spp. used as human and animal probiotics. Interestingly, *B. cereus* exhibits virulence in a strain specific manner. The pathogenic characteristic depends over strain and variety specific production of several extracellular factors viz. (phospholipase, cereulide (emetic toxin), enterotoxin Hbl, non-haemolytic toxin (Nhe), haemolysin IV) having role in cellular membrane disruption and induction of necrotic enterocolitis cytotoxin (Berthold-Pluta et al., 2015). Toxicity of virulent forms of *B. cereus* had been linked to the expression of plcR gene, that codes for most of the extracellular virulence factors (Hbl, Nhe, cytK) and proteins (Ramarao and Lereclus, 2006). Recently, data obtained from draft genome assemblies of 25 *B. cereus* strains clearly indicated sub-division of *B. cereus* into seven phylogenetic groups. Frequent horizontal transfer of pathogenicity factors among *B. cereus* has been proposed as an important factor determining the distribution of *Bacillus* spp. among pathogenic, non-pathogenic and probiotic species. The enterotoxin operons *viz*. nheABC and hblCDAB are chromosomally located and are abundant within *B. cereus*, which along with possibility of horizontal transfer raises safety concerns (Bohm et al., 2015).

These studies have revealed the phylogenetic relations among important species of *Bacillus* genus and are also indicating toward the necessity of more profound process for the selection and identification of *Bacillus* probiotic candidates. In this way, the complete genome sequencing and comparative analysis of various close relative *Bacillus* species of different groups has been provided by different research groups (Rey et al., 2004; Kolsto et al., 2009; Earl et al., 2012; Jeong et al., 2016; Krawczyk et al., 2016; Fan et al., 2017). The data of deeply sequenced *Bacillus* species from different sources can be utilized for the accurate identification and characterization of candidate probiotic strains. This may include characterization using advanced molecular techniques (such as PFGE, 16S rDNA sequencing, AFLP and MLST etc.).

## *Bacillus*: Probiotic Attributes In Gut

*Bacillus* spp. are preferentially aerobic to facultative aerobic (Hoffman et al., 1995), germination and outgrowth of spores in the intestine seem difficult to envisage because the mammalian intestine is an anaerobic environment. In order to qualify as a potential probiotic candidate, the *Bacillus* strains must possess the primary requirement of GIT stress tolerance, besides having good adhesion and bio-therapeutic properties (Thakur et al., 2016). Survival under the GIT stress represents another challenge for safe transit and localization in the gut. Bacilli are normally considered soil organisms however, number of them including *B. subtilis* have been reported in feces and ileal biopsies of volunteers (Fakhry et al., 2008). In this regard, Hong et al. (2009) reinforced a growing view that *B. subtilis* and probably other species had adapted to life within the human GIT, including ability to form biofilm, sporulate anaerobically and produce antimicrobials, and should be considered gut commensals, rather than solely soil microorganisms.

Earlier, Hyronimus et al. (2000) evaluated thirteen spore forming lactic acid bacilli for their resistance to acid and bile salt. *B. laevolacticus* DSM 6475 and *B. racemicus* IAM 12395 were found to tolerate pH 2.5 for up to six hours. Also, *B. racemilacticus* and *B. coagulans* strains were reported to be tolerant to bile concentrations over 0.3% (w/v). This study indicated high acid tolerance of *Bacillus* spp., however, the same was questionable for bile tolerance for few strains. In a recent study, twenty healthy European subjects were administered orally with Enterogermina^®^ containing spores of four strains of *B. clausii*, which could survive transit through the human GIT, during which germination, out-growth and multiplication could happen (Ghelardi et al., 2015). Similar to non-spore forming *Lactobacillus* spp., the survival rate of spore forming bacilli is also strain specific. Additionally, food matrix also plays an important role in survival of probiotics during simulated gastric juice conditions (Karu and Sumeri, 2016).

Different *Bacillus* strains have been reported to display antimicrobial, anti-oxidative and immune-modulatory activity in the host. The elements behind beneficial attributes of *Bacillus* spp. probiotics are explored in various studies, wherein these activities were found connected to their ability to produce antimicrobial peptides, small extracellular effector molecules and their ability to interact with host with the help of adhesion and attachment features (Khochamit et al., 2015). The antagonistic activity of *Bacillus* spp. has been explored against large number of pathogens. A study by Pinchuk et al. (2001) also reported the anti *H. pylori* activity of tested probiotic *B. subtilis* strains, which was attributed to the secretion of aninocoumacin A antibiotic. The antagonistic activity of aninocoumacin A was also documented against enteric *E. faecium* and *Shigella flexneri.* An interesting communication by Ripert et al. (2016) revealed that the probiotic *B. clausii* O/C strain protected vero and Caco-2 cells from the cytotoxic effect of *Clostridium difficili* and *B. cereus* toxins. This activity was mediated by serine protease(s) of *B. clausii*. On the other hand, exclusion of pathogen by the inhibition of bacterial biofilm is another potential attribute proposed for *Bacillus* strains. In this context, Gobi et al. (2016) demonstrated that the cell free extracts of *B. licheniformis* Dahb1 decreased the microbial adhesion to hydrocarbon, exopolysaccharide and biofilm metabolic activity, and decreased biofilm formation by the virulent *V. parahaemolyticus* Dahv2, in Asian catfish.

Recently, a bacteriocin producing strain of probiotic *Bacillus coagulans* had been isolated from traditional fermented fish of Manipur, India. The purified bacteriocin of low molecular weight displayed broad spectrum of antimicrobial activity against food borne and related clinically relevant pathogens, besides having lower cytotoxicity (Abdhul et al., 2015). Earlier, Joseph et al. (2013) also isolated and characterized bacteriocin producing strain of *B. subtilis* and displayed high level of antimicrobial activity of partially purified bacteriocin against foot ulcer bacterial pathogens, with highest recorded against *Klebsiella* spp. Such bacteriocin producing strains of *Bacillus* spp. have potential to be introduced as food biopreservative and as an antimicrobial in human and animal infections. In spite of pathogenic status of *B. cereus*, this species has also been explored for probiotic attributes. For example, *B. cereus* var. vietnami which used in Biosubtly^DL^ was found to produce bacteriocins like activity against different *Bacillus* species and it was shown to persist the mouse GIT up to 18 days. However, it was also found to produce enterotoxins (Duc et al., 2004). In addition, *B. cereus* var. toyoi, *B. cereus* YB-2, *B. cereus* G19 and *B. cereus* BC-01 were studied for their beneficial effect on livestock including rabbit, piglets and sea cucumber (Trocino et al., 2005; Altmeyer et al., 2014; Li et al., 2015).

## Health Benefits of Probiotic Spore Formers

In the food sector, the global trend is to incorporate probiotics into food matrix in order to provide some health-promoting component(s) beyond its traditional nutrients (Butel, 2014). *Lactobacillus* and *Bifidobacterium* spp. have earned many health claims, including their immune-modulation (Shida and Nomoto, 2013; Maneerat et al., 2014), anti-mutagenic (Sah et al., 2014), lowering of plasma triglycerides (Guo et al., 2013; London et al., 2014) and the potential to modulate host physiology via direct and indirect means (Di Caro et al., 2005; Fujiya et al., 2007). On contrary, there are few published reports dealing with the health benefits of probiotic spore formers (**Figure 1**). Efficacy of *Bacillus* spp. as probiotic has been screened in several *in vitro* and *in vivo* animal models and a few have also been validated in human clinical trials. The available data from these studies has presented the beneficial effects of different strains of *Bacillus* spp. for human health. For example, several researchers have recognized the preventive role of *Bacillus* probiotic in gut physiology impairment conditions (Lopetuso et al., 2016; Zhang et al., 2016). The amelioration of dysbiosis and gut inflammation by probiotic *Bacillus* strains was established by the ability of balancing gut flora toward beneficial microbial population and associated anti-inflammatory agents which helped to recover intestinal mucosa from illness generated injuries. Recently, an *in vivo* study by Haldar and Gandhi (2016) revealed that the oral administration of skim milk containing *Bacillus coagulans* B37 and *Bacillus pumilus* B9 decrease coliform counts in feces of treatment groups. Besides, the beneficial effect of *B. coagulans* on the gut metabolism is also evaluated by Lee et al. (2016), who reported that feeding of *B. coagulans* along with soya pulp to cholic acid fed rats, suppressed the production of secondary bile acid, improved intestinal permeability and reduced the bactericidal effect of bile acid which supported the growth of beneficial microbiota into the intestine. An exopolysaccharide (EPS) producing strain of *B. cereus* SZ-1 delayed DNA damage, increased cell survival and glutathione and catalase expression in H_2_O_2_ induced PC12 cells. The purified EPS from *B. cereus* could be useful for preventing oxidative DNA damage and cellular oxidation in pharmaceutical and food products (Zheng et al., 2016). The protective effect of *Bacillus* probiotics against toxins has also been reported by Ripert et al. (2016). In this interesting communication, authors perceived involvement of secreted serine proteases from *B. clausii* in normalization of toxin produced by *Clostridium difficili* and *B. cereus*. The purified component also exhibited antitoxic potential on Vero cell line treated with cell culture supernatants of both pathogens. Similarly, an antimicrobial peptide, antilisterial Subtilosin A derived from *Bacillus tequilensis* FR9 was also reported to exhibit pathogen invasion protection ability in HCT-116 human colon carcinoma cell line (Rani et al., 2016). In contrast, the bioavailability of nutraceuticals has seen to be increased in the presence of *Bacillus* strains. For example, Dudonné et al. (2015) has shown increased microbial degradation products of native cranberry phenolic compounds in *B*. *subtilis* CU1 supplemented rats.

The positive effect of *Bacillus* spp. to distant cells, beyond GIT has also been established by several researchers. In this context, Foligné et al. (2012) studied the effect of probiotic *Bacillus subtilis* PB6 on cytokine release profile of human immunocompetent peripheral blood mononuclear cells (PBMC). Strain PB6 induced substantial levels of IL-10 but very low levels of IL-12, TNFα, and IFNγ on human PBMC. In an interesting study, Abhari et al. (2016) demonstrated that oral administration of *B. coagulans* and inulin combination could improve the biochemical and clinical parameters of rheumatoid arthritis in rat model. Briefly, pre-treatment with the symbiotic diet significantly inhibited the fibrinogen, serum amyloid A and TNFα production, and significantly inhibited the development of paw swelling induced by complete Freund’s adjuvant. The immune-modulatory potential of probiotic *B. cereus* for livestock has also been investigated by few researchers. In an animal study, dietary supplementation of *B*. *cereus* var. toyoi to sows and piglets resulted in its recovery from feces, however, interestingly probiotic was detected in piglet feces before its dietary intake, indicating a secondary route of its intake besides diet. Dietary intake of *B. cereus* var. toyoi reduced the incidence of liquid feces and diarrhea (Taras et al., 2005). Additionally, the positive effect of *B. cereus* var. toyoi on secretion of interferon gamma and IL-4 and natural killer receptor 2D (NKG2D) expression in intraepithelial CD5+ γδ T cells of piglets has been suggested by few workers (Schierack et al., 2007; Altmeyer et al., 2014). Additionally, components secreted by potential probiotic *Bacillus* strains also possess anti-cancer activity. In an important analysis by Lee et al. (2012), surfactin like compound of *Bacillus subtilis* CSY191 could inhibit the growth of MCF-7 human breast cancer cells in a dose-dependent manner. Furthermore, the health benefits of *Bacillus* probiotic strains have also been proven in human subjects of different health and age groups. Some of the important clinical trials demonstrating the impact of spore-former probiotic *Bacillus* strains have been highlighted in **Table 3**.

**Table 3:** Clinical trials of probiotic *Bacillus* strains representing health benefits on human subjects.

Strain	Study	Outcomes	Reference
*B. coagulans* Unique IS-2	Phase II clinical study upon 28 patients with acute diarrhea	– Mean values for duration of diarrhea decreased	Sudha and Bhonagiri, 2012
		– Frequency of defecation was decreased	
		– Abdominal pain decreased	
		– Consistency of stool improved in treatment group	
*B. subtilis* 3 and *B. licheniformis* 31	Single-center, randomized, double-blinded, placebo-controlled clinical trial on 574 patients suffering from antibiotic-associated diarrhoea (AAD)	– The incidence of AAD and adverse effects related to the use of antibiotics in treatment group were significantly decreased	Horosheva et al., 2014
*B. coagulans* (Colinox)	Monocentric double-blind, placebo-controlled parallel group study on 52 adult subjects suffering from IBS	– Significant reduction of the bloating, discomfort and pain in Colinox group compared to placebo group	Urgesi et al., 2014
*B. coagulans* GBI-30 and 6086	Double-blind placebo-controlled trial on 17 HIV-1 infected persons	– The probiotic was safe and well tolerated	Yang O.O. et al., 2014
		– Appeared to improve chronic gastrointestinal symptoms	
*B. subtilis* CU1	Randomized, double-blind, placebo-controlled,	– Fecal and salivary secretory IgA concentrations significantly increased compared to the placebo	Lefevre et al., 2015
	parallel-arms study on 100 healthy subjects aged 60–74	– Frequency of respiratory infections in the probiotc group decreased	
*B. coagulans* GBI-30 and 6086	Double-blind, placebo-controlled crossover design on 36 healthy volunteers aged 65–80 years	– Significantly increased populations of *Faecalibacterium prausnitzii* and	Nyangale et al., 2015
		– Peripheral blood mononuclear cells (PBMCs) showed increase in the anti-inflammatory cytokine IL-10 after stimulation with LPS	
*B. clausii*	Double-blinded, placebo-controlled, randomized trial in 244 preterm neonates	– No significant difference in the incidence of late-onset sepsis (LOS), however, full feeds were achieved significantly faster in the probiotic group	Tewari et al., 2015
*B. coagulans* MTCC 5856	Double blind placebo controlled multi-centered trial in 36 diarrhea predominant IBS patients	– Decrease in the clinical symptoms like bloating, vomiting, diarrhea, abdominal pain and stool frequency	Majeed et al., 2016
		– Disease severity decreased and the quality of life increased	
*B. coagulans* GBI-30 and 6086 with casein protein	Placebo and diet-controlled study in 29 healthy male subjects	– Significantly increased post exercises perceived recovery and decreased muscle soreness	Jager et al., 2016
		– Showed a trend toward reduced muscle damage	

Besides having direct effect over host health, strains of *Bacillus* are currently also being employed for protective and therapeutic effect against several systemic clinical syndromes particularly, metabolic disorders. Several studies have established that natural products involving *Bacillus* spp. can be an alternate, safe and cost effective therapy for the management of metabolic syndromes. In one such study, solid state fermentation of soybean with *B. amyloliquefaciens* resulted in production of 1-Deoxynojirimycin, a potent alpha-glucosidase inhibitor (Cai et al., 2017). On similar lines, 13 weeks of dietary intervention with *B. licheniformis* 67 fermented soybean paste significantly prevented obesity related parameters in diet induced obese C57BL/6J mice (Choi et al., 2016). Fermented soybean fed group displayed lower values for blood glucose, insulin, serum and hepatic lipid profile, and body weight compared with high fat diet control group. The anti-obesity activity was attributed to the production of poly gamma glutamic acid by selective strains. The anti-diabetic functionality of *B. licheniformis* fermented soybean had also been attributed to the reduced accumulation of beta amyloid in brain hippocampus, preventing beta amyloid mediated insulin resistance and beta cell death. The study displayed glucose homeostaisis effects of fermented soybean in diabetic rats with experimental Alzheimer’s type dementia (Yang H.J. et al., 2014). Similarly, Purified exopolysaccharide from *B. subtilis* suppressed cardiovascular disease related parameters in streptozoticin induced diabetic rats (Ghoneim et al., 2016). In this study, the therapeutic effect of EPS was recorded due to reduced blood glucose, troponin, total serum cholesterol, LDL, and VLDL as well as suppression of ICAM and VCAM expression. Earlier, Zouari et al. (2015) also explored the anti-diabetic and anti-lipidemic properties of biosurfactant produced by *B. subtilis* SPB1 strain in alloxan induced diabetic rats. Upon oral administration, the biosurfactant reduced the plasma alpha-amylase activity and rendered protection to pancreatic beta cells. Besides displaying hyperglycemic effects, biosurfactant administration regulated serum lipid profile by promoting HDL-cholesterol and delaying the absorption of LDL-cholesterol and triglycerides. Aforesaid studies clearly support the rich bio-therapeutic potential of spore forming *Bacillus* strains, which further needs validation in human clinical trials. In light of the above reports, it can be stated that the candidate probiotic *Bacillus* strains themselves or their metabolites could also be considered as a potential candidate for management of metabolic disorder.

## Probable Mechanism of Action

**FIGURE 4:**
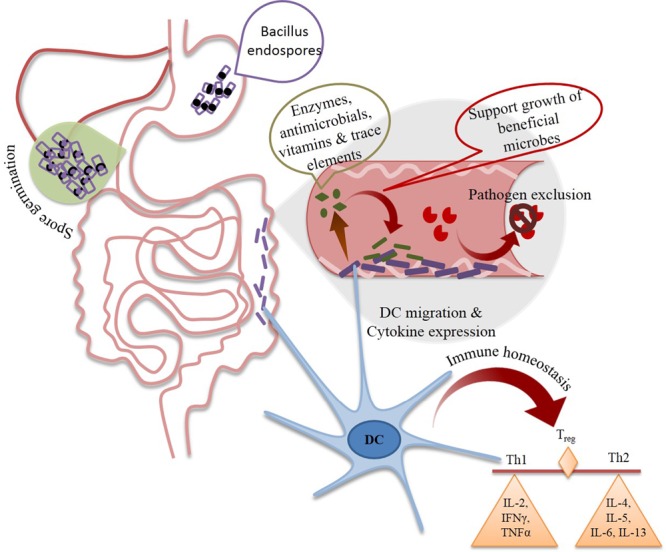
Different possible mechanisms of health benefits of spore forming *Bacillus* probiotics (SFBP). Daily intake of SFBP is resulted in increased their colonization in gut resulting in increased number of beneficial microbial population and decreased number of pathogenic strains. Moreover, SFBP could proliferate different immune cell for production of anti-inflammatory cytokines to maintain immune homeostasis. DC, Dendritic cells, T_reg_, regulatory T cells, Th, T helper cell, IL, interleukin, IFNγ, interferon γ, TNFα, tumor necrosis factor α.

Several mechanistic studies have attempted to underline the probable mechanism of action of candidate probiotic *Bacillus* strains. The mechanisms by which spore forming probiotics (SFP) could enhance health of the host include stimulation of immune system, synthesis of different antimicrobials, like bacteriocins, enzymes and modulation of the composition of gut microbiota (**Figure 4**). The mechanism behind establishment of gut homeostasis involves promotion of growth of other beneficial microbes and suppression of pathogen and pathogen induced inflammatory response of intestinal mucosa. Microbial interference therapy depends on production of antimicrobial(s) by different probiotic strains. *Bacillus* spp. are known to produce several antimicrobial substances, e.g., bacteriocins, bacteriocins like inhibitory substances (e.g., Subtilin and Coagulin) and antibiotics (e.g., Surfactin and Bacilysin). *B. subtilis* var. natto has also been shown to inhibit the growth of *Candida albicans* in the intestinal tract, which is attributed to production of antibiotic, Surfactin, having activity against yeast (Ozawa et al., 1979; Nagal et al., 1996). In a recent study, *B. subtilis* R0179 was shown to have significant inhibitory effect on the growth of *Candida* spp. proposing it as an alternate therapy against oral candidiasis (Zhao et al., 2016). Also, Biosporin^®^ containing *B. subtilis* 2335 and *B. licheniformis* 2336 (Biofarm, Ukraine) had been applied as probiotic supplement for humans and it possess antibacterial activity against *Heliobacter pylori* (Pinchuk et al., 2001). On the other hand, the positive influence of probiotic *Bacillus* strains on the growth and composition of commensal and beneficial species on gut could be mediated by the production of extracellular enzymes, vitamins and peptides. The impact of probiotic strains on host physiology is also a major factor behind maintenance of gut homeostasis. A comprehensive study in human subjects using DNA microarray technique observed that the genes involved in inflammation, immune response, defense response, intestinal permeability, cell adhesion, cell growth, cell differentiation, cell signaling, apoptosis, signal transcription, and transduction in intestinal mucosa were modulated upon *B. clausii* consumption (Di Caro et al., 2005). Surface-associated proteins from vegetative cells and spores of *B. cereus* might play an important role in interaction between these strains within human GIT (Sanchez et al., 2009). In addition, Fujiya et al. (2007) has also explained the role of quorum sensing molecules (QSMs) secreted by *B. subtilis* strain JH 642 in preservation of intestinal health. In this study, authors found that quorum sensing pentapeptide, competence and sporulation factor of *B. subtilis* JH 642 is involved in organic cation transporter mediated activation of p38 and MAPK pathways in Caco2_bbe_ cells. This interaction serves an example of probiotic mediated change in behavior of host and composition of colonic flora.

The impact of probiotic *Bacillus* strain has also been reported on distal organs. For instance, the spores of *B. clausii* (Enterogermina^®^) were shown to enhance the production of IFN-γ in murine spleen cells, rabbits and mice animal models (Muscettola et al., 1991, 1992; Kosak et al., 1998). Vegetative cells of *B. firmus* have been shown to stimulate the proliferation of human peripheral blood lymphocytes *in vitro* (Prokesova et al., 1994). Also, spores of *B. subtilis* PB6 from Anaban^TM^ had displayed anti-inflammatory effect in mice models that has been observed to be mediated through the modulation of IL-10, TNF-α and IFN-γ expression (Foligné et al., 2012).

## Safety of Probiotic Spore Formers

Safety of a food product signifies the absence of any notable adverse health effects upon consumption under defined conditions (Kalliomäki et al., 2001). Probiotics are recognized for their long history of safe use. However, consumption in large amounts under immune compromised state may raise several safety concerns. Among *Bacillus* spore-formers, *B*. *anthracis* and *B. cereus* are well known pathogens. In case of *B. cereus*, pathogenicity varies from case to case; with some strains being the carriers for enterotoxin genes (Rowan et al., 2001). The occurrence of *Bacillus* spp. in food does not always cause foodborne illnesses or food spoilage; some species likewise, *B. subtilis* are used for preparation of East Asian fermented foods such as natto (Hosoi and Kiuchi, 2003). *B. subtilis* causes food-borne illnesses, with different symptoms, mainly vomiting (Kramer and Gilbert, 1989). At least one *B. subtilis* strain carries all three genes required to produce the Hbl enterotoxin normally produced by *B. cereus* (Rowan et al., 2001). On the other hand, *Bacillus subtilis* ATCC 6633 is known to produce a wide variety of antibacterial and antifungal compounds (Stein, 2005).

Furthermore, *Bacillus* spp. are used widely in transformation, whereas the plasmid encodes conjugative or mobile elements (Mullany et al., 2004). The commercial *B. cereus* IP5832 (Bactisubtil^®^) was isolated from the stools of patients with diarrhea (Kniehl et al., 2003). The strain was later shown to carry genes coding for endotoxin (Duc et al., 2004). Toyocerin^®^ (Asahi Vet S.A., Tokyo, Japan), containing *B. cereus* var. toyoi, is licensed in EU for animal feed (reviewed in Cutting, 2011). In market, several probiotic *Bacillus subtilis* based products like BioGrow^®^, mixture of *B. subtilis* and *B. licheniformis* (Provita Eurotech Ltd, Omagh, UK); BioPlus^®^2B, mixture of *Bacillus* spp. (CHR. Hansen, Hoersholm, Denmark, EU approved); and AlCare^TM^, containing *B. licheniformis* (Alpha-pharmaInc, Melbourne, VIC, Australia, not licensed in EU) are being used for animal feed and aquaculture.

In South East Asia, different probiotic products containing *Bacillus* stains, either as single or mixed with other *Lactobacillus* strains, are used as an alternative to conventional antibiotics. These strains are marketed as antibiotic resistant probiotics. Therefore, there is a risk of transferring antibiotic resistance genes to commensal and pathogens in gut of humans and animals and release of drug resistance genes to the environment through feces (SCAN, 1999, 2003). Enterogermina^®^ contains spores of four strains of *B. clausii*, which are resistant toward chloramphenicol and tetracyclin. Suspension of these spores (2 × 10^9^cfu/ml) is used as medical supplement along with antibiotics against infantile diarrhoea (Green et al., 1999; Senesi et al., 2001).

Esporafeed^®^ Plus, a feed additive containing *B. cereus* strain carrying a plasmid borne *tet*B gene was withdrawn from use in different European countries (SCAN, 1999). Also, due to concern of transfer of erythromycin resistance, AlCare^TM^ was considered unsafe for feeding pigs (SCAN, 2002). In Vietnamese market, several *Bacillus* spp. products have been applied and licensed for human use such as, Biobaby^®^ (ILdongPharma Co., Ltd., Korea), which contains three different spore forming probiotics, including *B. coagulans* (incorrectly, *Lactobacillus sporogenes*, widely used in India and one strain that has been granted as GRAS by FDA in the United States), *B. subtilis* and *C. butyricum*. Also, several *in vitro* and *in vivo* studies were performed in order to examine the toxicity of different species including *B. subtilis* var. natto and *Bacillus indicus* (Hong et al., 2008), *B. licheniformis* 2336 (Sorokulova et al., 2008), and *B. coagulans* (Endres et al., 2009). All appear to show no indications of adverse effects indicating the most occurrence of illness associated with *Bacillus* probiotic strains supplement of humans are result of either opportunistic infections or miss-diagnosis (reviewed in Cutting, 2011). Recently, Zhu et al. (2016) evaluated the safety of 15 commercial probiotic *B. cereus* products in China, they found that these products represent a potential risk for public health in China because and they recommended stricter safety regulation for these products is needed.

## Regulatory/Legislative Status

The “health claims” of commercially available bio-therapeutics are allied to the viability, activity and composition of component microorganisms. Likewise, the entitlements of probiotic products being marketed as functional food, dietary supplement or drug are also elucidated by the state of probiotic strains. Thereby, the quality of commercial probiotic products is an important issue to be considered for regulation. Several researchers have identified discrepancies between labeled and actual contents of commercial probiotic products (Weese and Martin, 2011; Drago et al., 2013). Viability of probiotics, misidentifications at the genus/species level (marking *Lactobacillus sporogenes* in place of *B. coagulans* is a common example) and cross contamination by microorganisms are the main non-conformities during the evaluation of the quality of commercial probiotics. Consequently, despite the fact that significant amount of scientific literature is being produced about specific clinical benefits of probiotic microorganisms, there is an increasing demand for the legislative regulation on manufacturing practice, labeling and advertising of probiotic products.

Thereby some countries are developing their regulatory system to warrant the safety and wellbeing of consumers. In Japan, Food for Specified Health Use (FOSHU) system has defined functional food as ‘processed foods containing ingredients that aid specific bodily functions in addition to being nutritious’. The country has also regulation on the prevention of mislabeling of such foods. European countries have Food Products Directive for the regulation of food labeling. Probiotic foods also come under the regulation of this law. According to Directive, the contents in labeling must not be misleading for the purchasers. In UK, Joint Health Claims Initiative (JHCI) defines a health claim as ‘a direct, indirect or implied claim in food labeling, advertising and promotion that consumption of a food carries a specific health benefit or avoids a specific health detriment’. In United States, FDA has approved 12 health claims for foods. In addition, a joint FAO/WHO expert consultation on health and nutritional properties of probiotics in food have given scientific recommendations about the characterization, safety, efficacy, and labeling of the probiotic products. The report of this consultation also stated that the regulatory status of probiotics is not well established on an international basis (FAO/WHO, 2001). Thereby experts of consultation also made a number of recommendations pertaining to regulatory matters which will help to improve the regulatory status of functional food in global market.

## Current Global Status

The probiotic market share is valued at the size of USD 36.6 billion in 2015 which will be widened with over 7% CAGR growth from 2016 to 2023^2^. The Asia pacific will make the largest industry participant as they accounted for more than 40% of the global industry. India, China, and Japan are the major contributing factors in this field while both India and China will also see maximum growth in upcoming years^3^. Growing awareness about health, lifestyle, and increasing issues related to metabolic and digestive disorders are important contributing factors in the hike in probiotic market share. Moreover, the presence of international companies is also increasing the attention of consumers toward this health promoting supplements. If we look by the types of organisms, the probiotic market is categorized into five groups, i.e., *Lactobacillus, Bifidobacterium*, spore formers, yeast and others. Hence, the spore former probiotics are making a major contribution in global nutraceutical as well as pharmaceutical market.

## Challenges Ahead

Probiotics enjoy the GRAS status and are freely consumed globally without any safety concern. Their efficacy and safety has been demonstrated in several *in vitro, in vivo* and human clinical trials. However, few recent studies have raised concern about their safety and dose in immune-compromised people (Redman et al., 2014). Although *Bacillus* probiotics have an overall excellent health promoting record, especially in preventing and curing of diarrhoea, giggivitis, *H. pylori* infection and maintaining homeostasis of intestine (Khodadad et al., 2013; Lefevre et al., 2015; Alkaya et al., 2016; Lopetuso et al., 2016). Their application with certain immune deficient population especially for critically ill, neonates and elderly groups should be evaluated and regulated carefully since reports related to bacteremia in immune-compromised patient treated with spore former and other probiotics has been recorded repetitively (Doron and Snydman, 2015). However, the importance of identification to strain level is also important to detect and eliminate any causal link between probiotics and strains isolated from immune-compromised hosts. Thereby, it is important to keep in mind that clinical trials of these formulations should cover the sufficient ratio of target population including people with low immunity.

## Author Contributions

FE, NR, and HP has drafted the manuscript. FE, NR, RG, CS, and HP equally contributed in writing of manuscript. HP did the editing of manuscript.

## Conflict of Interest Statement

The authors declare that the research was conducted in the absence of any commercial or financial relationships that could be construed as a potential conflict of interest.

## Footnotes

1 http://www.bacterio.net/bacillus.html

2 https://www.gminsights.com/industry-analysis/probiotics-market

3 http://www.grandviewresearch.com/industry-analysis/probiotics-market

## Abbreviations

GIT: gastrointestinal tract
GRAS: generally recognized as safe
ITS: internal transcribed spacer
LAB: lactic acid bacteria
PBMC: peripheral blood mononuclear cells
SFP: spore forming probiotics.
